# A correspondence on “Impact of pecto-intercostal fascial block on postoperative fatigue in elderly patients undergoing off-pump coronary artery bypass grafting: a randomized clinical trial”

**DOI:** 10.1097/JS9.0000000000003099

**Published:** 2025-07-17

**Authors:** Weikai Dong, Wei Li

**Affiliations:** Department of Cardiovascular Surgery, Binzhou Medical University Hospital, Binzhou, Shandong, China

*Dear Editor,*

We found the recently published article “Impact of pecto-intercostal fascial block on postoperative fatigue in elderly patients undergoing off-pump coronary artery bypass grafting: a randomized clinical trial^[1]^” to be of great interest. According to this study, the administration of pecto-intercostal fascial block (PIFB) is effective in lowering postoperative fatigue syndrome (POFS) and improving the recovery of elderly patients undergoing off-pump coronary artery bypass grafting (CABG). Notwithstanding, we would like to point out several methodological considerations and areas that need more fine-tuning.

Firstly, the study focuses on the early postoperative period (up to 7 days) and lacks long-term follow-up data beyond 8 weeks^[2]^. POFS may last for months in some patients, particularly those who undergo major cardiac surgery. An extended follow-up period would be necessary to evaluate any long-term effects of PIFB on the occurrence and recovery from POFS. Such extended observation would clarify whether the benefits observed during the early postoperative phase are maintained over time.

Secondly, the study utilizes a single-injection technique for PIFB. Despite its quite beneficial short-term effects, it may fail to deliver prolonged analgesic effects and sustained recovery advantages. Ongoing use of PIFB or PIFB in multimodal analgesia may offer more enduring pain relief^[3]^ and a further reduction in the incidence of POFS. Future studies should explore these alternative strategies to improve the clinical effectiveness and utility of PIFB.

Thirdly, the study population is restricted to elderly patients undergoing off-pump CABG. Although this population is a clinically important high-risk group for POFS, the present results may not apply to younger patients, those undergoing on-pump CABG^[4]^ or patients receiving other forms of cardiac or thoracic surgery. Including a wider array of surgical procedures and patient demographics would likely improve the external validity of our results.

Fourthly, although the study notes the absence of adverse events of PIFB, safety has not yet been fully established in larger and more diverse populations. Further studies should be conducted to assess the long-term safety and efficacy of thoracic wall fascial blocks like PIFB for cardiac surgery^[5]^.

Lastly, the study fails to identify the mechanisms through which PIFB might reduce POFS. While the authors suggest that reduced pain, opioid consumption, and inflammatory response may lead to the observed beneficial outcomes, further mechanistic studies are needed to confirm this.

In short, this study shows that PIFB can mitigate early POFS and expedite recovery among elderly patients undergoing off-pump CABG. However, addressing the above limitations can enhance its clinical applicability and significance. Further studies are required to optimize PIFB implementation and improve postoperative outcomes following cardiac surgery by extending the follow-up period, evaluating different PIFB techniques, including various patient cohorts, and studying the underlying mechanisms.

## Declaration of AI use in the research and manuscript development

In compliance with the TITAN Guideline 2025^[6]^ for transparent reporting of Artificial Intelligence (AI) in research and manuscript development, we hereby declare that no AI tools or technologies were employed at any stage of the research process or in the preparation of this manuscript. All aspects, including the formulation of research questions, data analysis, writing, and revisions, were carried out exclusively by the authors without reliance on AI systems. The content of this manuscript is entirely the result of human intellectual effort and expertise, and the authors assume full responsibility for its accuracy and integrity. This declaration is made to ensure transparency and adherence to the standards outlined in the TITAN Guideline 2025.

## Data Availability

Not applicable.
